# Indications for Surgical Resection in Patients With Neuroendocrine Tumor Liver Metastases: An Intensive Surgical Experience of a High‐Volume Center

**DOI:** 10.1002/ags3.70082

**Published:** 2025-08-27

**Authors:** Daisuke Asano, Toshitaka Sugawara, Keiichi Akahoshi, Shotaro Gan, Shohei Motohashi, Shuichi Watanabe, Yoshiya Ishikawa, Hiroki Ueda, Atsushi Kudo, Daisuke Ban

**Affiliations:** ^1^ Department of Hepatobiliary and Pancreatic Surgery Institute of Science Tokyo Tokyo Japan; ^2^ Division of Surgical Oncology, Department of Surgery University of Colorado School of Medicine Aurora Colorado USA

**Keywords:** indication, NELM, neuroendocrine neoplasm, neuroendocrine tumor, surgery

## Abstract

**Background:**

Surgical resection for neuroendocrine liver metastasis (NELM) is the key to long survival; however, the indications remain unclear due to the high recurrence rate. We aimed to identify candidates who would benefit from surgical resection for NELM.

**Methods:**

Patients with NELM treated at our institution from January 2005 to December 2020 were included. Neuroendocrine carcinoma (NEC) was excluded. Risk factors for overall survival (OS) and recurrence‐free survival (RFS) were analyzed. The cut‐off value for the number of NELM predicting poor RFS was determined by minimum *p*‐value approach.

**Results:**

Of the total 126 patients, 67 patients underwent liver resection. The median follow‐up time from the date of initial diagnosis of NELM was 4.3 years. Surgical resection and NET‐G1/2 were associated with good OS in multivariate analysis (*p* < 0.001). In patients underwent R0/1 resection (*n* = 44), NET‐G3 [HR: 3.1 (95% CI 1.4–7.2)] and the number of NELM [HR: 1.1 (95% CI 1.0–1.1)] were associated with poor RFS in multivariate analysis. The optimal cut‐off value for the number of NELM was calculated as 8. The median RFS for patients with 8 or more liver metastases or NET‐G3 was 3.9 months, which was extremely short compared to patients with NET‐G1/2 (13.8 months) and to those who had fewer than 8 liver metastases (19.1 months).

**Conclusion:**

This study suggests that fewer than 8 liver metastases and NET‐G1/2 are indications for surgical resection in patients with NELM considering the RFS. Surgical resection for patients with 8 or more liver metastases or NET‐G3 needs deliberate selection.

## Graphical abstract

In patients with NELM, those with < 8 liver metastases and G1/2 tumors are the favorable candidates for liver resection. The important point of this study is that the patients who do not meet the above criteria should be considered for multidisciplinary treatment because of their extremely shorter recurrence‐free period.

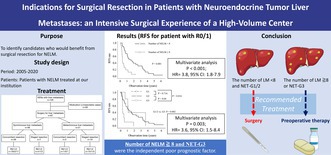

## 1 Introduction

Although neuroendocrine neoplasms (NENs) are literally rare, the number of patients with NENs has increased recently [1, 2]. The classification of grade 3 neuroendocrine tumor (NET‐G3), neuroendocrine tumors (NETs) with a well‐differentiated morphology and a Ki‐67 index higher than 20%, was first defined for pancreatic NENs in the World Health Organization (WHO) 2017 classification and used for gastrointestinal NENs in the WHO 2019 classification [3]. In contrast to the aggressive behavior of neuroendocrine carcinoma (NEC) [4], NETs are commonly reported to be indolent. However, approximately 40%–85% of patients with NET have synchronous or metachronous neuroendocrine liver metastasis (NELM), and many of which have a worse prognosis [5].

Surgical resection has been reported as the key treatment for long‐term survival in patients with NELM because of the paucity of effective drugs. Although it is still controversial [6, 7], some studies have shown the benefits of surgical treatment for this population in terms of overall survival (OS) [5, 8, 9, 10, 11]. Another role of resection is that it can provide a systemic therapy break for patients experiencing severe drug side effects. In this situation, surgical resection cannot be justified when a short recurrence‐free survival (RFS) is expected. Indeed, recurrence is a major problem; median RFS after resection of NELM ranges from 14 to 23 months [12, 13, 14, 15, 16], and the 1‐, 3‐, and 5‐year RFS rates have been reported to be 54.7%, 23.9%, and 13.4%, respectively [16]. While retrospective studies have reported survival benefits associated with surgery, such findings are often influenced by strong selection bias. Therefore, even in retrospective analyses, focusing on RFS may provide a more objective measure of surgical appropriateness. Optimal patient selection considering RFS is needed. To date, evidence regarding indications for surgical resection for patients with NELM is limited to several retrospective studies [13, 15, 16, 17, 18, 19, 20, 21, 22].

This study aimed to evaluate the effect of surgical resection on OS in patients with NELM as the primary outcome. In addition, RFS was analyzed as a secondary outcome in patients who underwent R0/1 resection, as RFS may provide an objective indicator for appropriate surgical indication.

## 2 Methods

### 2.1 Patients

Clinical data of patients with NELM who were treated at Institute of Science Tokyo Hospital (Tokyo, Japan) between January 2005 and December 2020 were retrospectively reviewed in a prospectively maintained database. Patients with NEC or who received only supportive care were excluded. All pathological findings were evaluated histologically by two certified pathologists, and consensus was reached by joint review in cases of disagreement. If the Ki‐67 value was calculated from both the primary tumor and metastases, the higher value was adopted. All NENs were pathologically classified in accordance with the WHO 2019 classification [3]. NET‐G3 was defined as a well‐differentiated tumor with a Ki‐67 index exceeding 20%. The number of liver metastases was determined pathologically. In R2 resection cases, however, the number of metastases was determined using preoperative imaging findings, as complete pathological assessment was not possible due to the presence of unresected tumors. All patients were identified from our institution's prospectively maintained database, which contains demographic, clinical, operative, pathological, and follow‐up data. This study conformed to the ethical standards of the World Medical Association (Declaration of Helsinki). Informed consent was obtained using an opt‐out approach, as approved by the institutional review board (Human Research Ethics Committee, Institute of Science Tokyo; approval ID: I2025‐097).

### 2.2 Treatments

According to the institutional protocol, patients with NELM were principally evaluated using transabdominal ultrasound, contrast‐enhanced CT, and Gd‐EOB‐DTPA‐enhanced magnetic resonance imaging (EOB‐MRI). Somatostatin scintigraphy and/or FDG‐PET were combined as needed. The treatment strategies were determined at a weekly multi‐disciplinary team conference. Surgical resection was performed when curative resection (in combination with ablation) could be achieved based upon pre‐operative studies. R1 resection was defined as the presence of microscopic residual tumor identified by pathological examination applying the 0 mm rule. R2 resection was defined as the presence of macroscopic residual tumor, indicating that part of the tumor was not resected. Preoperative therapy (POT) followed by resection was selected for patients with multiple metastases. The regimens for POT were sunitinib, everolimus, S‐1/streptozocin, capecitabine/temozolomide, and/or somatostatin analogs. POT was omitted in the following cases: patients with a small number of metastases, poorly‐controlled hormonal symptoms, or those who did not consent to undergo POT. Microwave ablation was combined with liver resection depending on the number and location of tumors. Adjuvant therapy was administered at the physician's discretion. Although there is little evidence for the use of somatostatin analogs in postoperative adjuvant therapy [23], somatostatin analogs were used for the postoperative adjuvant regimen because of their minimal side effects in our institutional experience.

Laboratory tests were performed for all patients at least every 3–6 months. Contrast‐enhanced CT or EOB‐MRI was also performed at the same interval. At least two radiologists diagnosed progression and relapse. OS and RFS were determined from the date of first diagnosis of NELM and the date of first hepatectomy, respectively.

### 2.3 Statistical Analysis

Categorical variables are reported as frequencies and percentages, and continuous variables are presented as medians and (interquartile) ranges. Survival curves were constructed using the Kaplan–Meier method, and differences in survival curves were compared with the log‐rank test. A Cox proportional hazard model with stepwise selection was used for the multivariate survival analysis. A univariate Cox proportional hazard model analysis was performed with all variables. Two‐sided *p*‐values were calculated, and differences were considered statistically significant at *p* < 0.05. The minimum *p*‐value approach [24] was applied to determine the cut‐off value for liver metastases based on the log‐rank test for the recurrence free survival after the first resection of NELM. X‐tile [25] software version 3.6.1 (Yale University) was also applied to obtain the optimal cut‐off value. OS was determined from the date of first diagnosis of NELM. SPSS Statistics version 29.0 (IBM Corp., Armonk, NY, USA) was used for all statistical analyses.

## 3 Results

### 3.1 Flow of the Treatment

**FIGURE 1:**
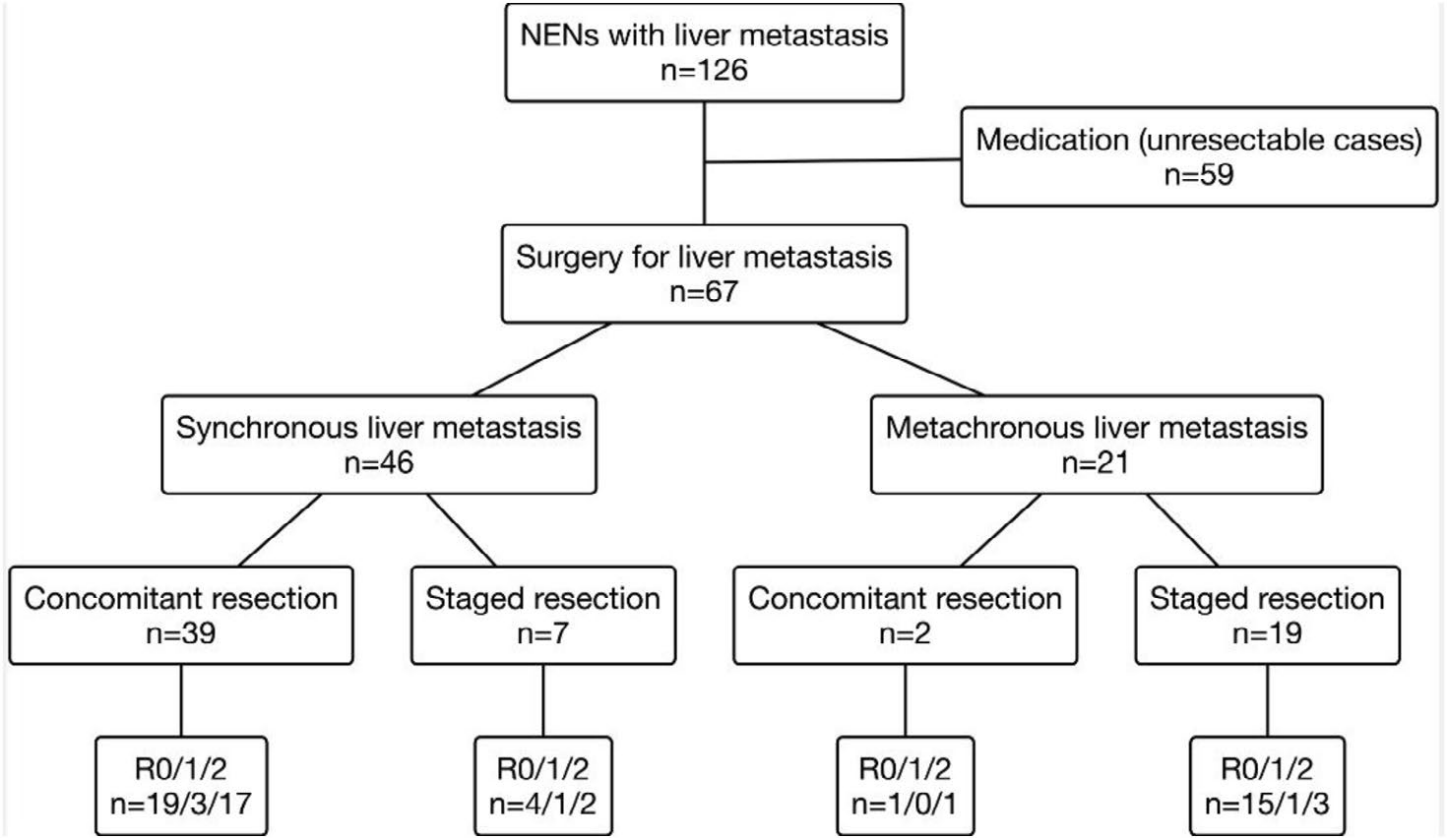
The treatment strategy in this study.

The treatment strategies are shown in Figure 1. A total of 126 patients with NELM were included and 67 of them underwent metastatic liver resection. The remaining 59 patients received non‐surgical treatment. Among 46 patients with synchronous liver metastasis, 39 patients underwent concomitant resection for primary and metastatic liver tumors, and 7 patients underwent metachronous liver resection after primary tumor resection; defined as staged resection. In contrast, 19 of 21 patients with metachronous liver metastasis underwent staged resection. Two patients underwent concomitant resection despite metachronous liver metastasis because the metastatic tumor was identified during observation of the primary tumor. Intraoperative microwave ablation was performed in 10 cases among R0/1 resection patients and 4 cases among R2 resection patients. POT was administered in 43 of 67 patients (64%) who underwent surgery for NELM. POT regimens prior to liver resection comprised sunitinib, everolimus, S‐1/streptozocin, capecitabine/temozolomide, and somatostatin analog monotherapy in 22 (51%), 7 (16%), 6 (14%), 1 (2%), and 7 (16%) patients, respectively. R0/1 resections were achieved in 44 patients (66%), while R2 resection was achieved in 23 patients (34%). The reasons for R2 resection included unexpected findings during surgery such as multiple liver metastases, unresectable status of the primary tumor, and extrahepatic distant metastasis including peritoneal dissemination. Additionally, a few R2 resections were intentionally performed in selected cases with limited bone or distant lymph node metastases, or in patients requiring surgical debulking to control severe hormone‐related symptoms.

### 3.2 Clinicopathological Characteristics of All Patients With NELM

The clinicopathological characteristics of all patients are shown in Table 1 (*n* = 126). The location of the primary tumor was the pancreas in 83 cases (66%). The median size of the primary tumor was 28 mm, and the median number of liver metastases was eight. The median Ki‐67 value was 10%. Patients with NET‐G1/2/3 were 23 (18%), 80 (64%), and 23 (18%), respectively. The number of patients with extrahepatic metastases, microwave ablation or without primary tumor resection was higher in the medication group than in the surgery group, but the other factors were not significantly different.

**TABLE 1:** Clinicopathological characteristics of all patients.

Variable	Total (*n* = 126)	Surgery for NELMs (*n* = 67)	Medication (*n* = 59)	*p*
Age (years)	57 (20–82)	52 (29–75)	59 (40–78)	0.428
Sex (male)	65 (52)	30 (45)	35 (59)	0.103
Primary tumor location				0.220
Pancreas	83 (66)	46 (69)	37 (63)	
Alimentary tract	38 (30)	17 (25)	21 (36)	
Unknown	5 (4)	4 (6)	1 (1)	
Primary tumor size (mm)	28 (16–40)	28 (16–37)	27 (20–43)	0.668
Maximal size of liver tumor (mm)	25 (12–53)	24 (10–57)	25 (13–52)	0.947
Number of liver metastases	8 (3–20)	6 (3–19)	10 (4–30)	0.065
Synchronous liver metastasis (yes)	86 (68)	46 (69)	40 (68)	0.918
Extrahepatic metastasis (yes)	19 (15)	4 (6)	15 (25)	0.002*
Resection of primary tumor (yes)	77 (61)	54 (81)	23 (39)	< 0.001*
Ki‐67	10 (5–17.6)	9.6 (5.5–17)	10 (3.5–18)	0.809
Grade				0.137
G1	23 (18)	8 (12)	15 (25)	
G2	80 (64)	45 (67)	35 (59)	
G3	23 (18)	14 (21)	9 (15)	
Surgical status				< 0.001*
R0/1	44 (35)	44 (66)	0 (0)	
R2	23 (18)	23 (34)	0 (0)	
Medication	59 (47)	0	59 (100)	
Microwave ablation (yes)	14 (11)	14 (21)	0	< 0.001*
Chromogranin A (+)	102 (81)	52 (80)	50 (88)	0.251
Synaptophysin (+)	115 (91)	62 (95)	53 (93)	0.569
NCAM (+)	94 (75)	53 (82)	41 (76)	0.454
Hereditary (yes)	6 (5)	3 (4)	3 (5)	0.598
Functionality (yes)	15 (12)	5 (7)	10 (17)	0.101

*Note:* Continuous variables are expressed as median (interquartile range).

Abbreviation: NCAM, neural cell adhesion molecule.

* *p* < 0.05 was considered statistically significant.

### 3.3 Survival Analyses of Patients With NELM

The median follow‐up time from the date of first diagnosis of NELM was 4.3 years (interquartile range: 3.3–6.4 years). The median survival time and 5‐year OS rates were 9.4 years (95% confidence interval [CI]: 5.7–13.2 years) and 69%, respectively.

**FIGURE 2:**
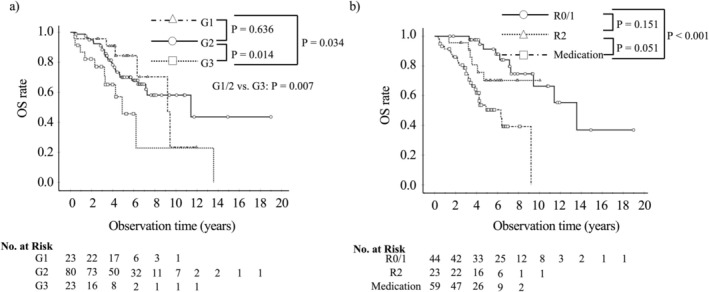
Comparison of OS by tumor grade (a) and surgical status (b). (a) Patients with NET‐G1 and G2 had comparable OS, however, those with NET G3 had significantly worse OS than those with NET‐G1 or G2. (b) Patients with R0/1 resection had better OS than those with medication.

Kaplan–Meier survival curves for each tumor grade and surgical status are shown in Figure 2. The median survival time (MST) for NET‐G1, G2, and G3 patients was 9.2, 11.4, and 4.9 years, respectively. The OS differed significantly between G1 and G3 (*p* = 0.034), G2 and G3 (*p* = 0.014), and G1/2 and G3 (*p* = 0.007). The NET‐G1 and NET‐G2 patients with NELM showed similar OS (*p* = 0.636). Patients who underwent R0/1 resection had a better prognosis than those who received medication (MST: 13.6 vs. 6.3 years; *p* < 0.001), and patients with R2 resection also tended to have better OS compared to medication group (*p* = 0.051).

Risk factors for OS are shown in Table 2. In univariate analysis, among the potential risk factors, tumor grade (G3) (*p* = 0.009), extrahepatic metastasis (*p* = 0.015), and surgical status (*p* < 0.001) were associated with OS. Multivariate analysis showed that both tumor grade (G3) and surgical status were independently associated with OS. Both R0/1 resection (*p* < 0.001; hazard ratio = 0.1, 95% CI: 0.06–0.3) and R2 resection (*p* = 0.01; hazard ratio = 0.3, 95% CI: 0.1–0.8) prolonged OS significantly longer than medication, and patients with NET‐G3 had a significantly worse prognosis than those with NET‐G1/2 (*p* < 0.001; hazard ratio = 4.0, 95% CI: 1.9–8.3).

**TABLE 2:** Prognostic factors for overall survival in all patients.

Prognostic factor	Univariate analysis		Multivariate analysis	
	Hazard ratio (95% CI)	*p*	Hazard ratio (95% CI)	*p*
Gender (male)	0.8 (0.5–1.5)	0.576		
Age	1.0 (1.0–1.0)	0.459		
Primary tumor location (pancreas)	1.0 (0.9–1.2)	0.839		
Tumor grade G1/2	Ref.			
G3	2.5 (1.3–5.1)	0.009*	4.0 (1.9–8.3)	< 0.001*
Maximal size of liver tumor ≥ 30 mm	1.6 (0.9–3.0)	0.128		
Synchronous liver metastasis	0.6 (0.3–1.3)	0.208		
Extrahepatic metastasis (yes)	2.5 (1.2–5.1)	0.015*		0.741
Number of liver metastases	1.0 (0.99–1.01)	0.671		
Surgical status		< 0.001*		< 0.001*
Medication	Ref.		Ref.	
R0/1	0.2 (0.07–0.4)	< 0.001*	0.1 (0.06–0.3)	< 0.001*
R2	0.4 (0.2–0.9)	0.036*	0.3 (0.1–0.8)	0.01*
Hereditary (yes)	1.6 (0.5–5.2)	0.442		
Functionality (yes)	1.1 (0.4–3.1)	1.083		

Abbreviation: CI, confidence interval.

* *p* < 0.05 was considered statistically significant.

Among 44 patients who underwent R0/1 resection, the median RFS was 12.3 months (95% CI: 7.0–17.6 months), and 22 of 44 patients (50%) had recurrence within 1 year from surgery. Four patients did not experience recurrence within the observation period. Patients who developed recurrence within 1 year (*n* = 22) experienced shorter OS compared with those with RFS > 1 year (*n* = 17) (*p* = 0.041). Furthermore, the OS of those with RFS ≤ 1 year was comparable to those with R2 resection (*p* = 0.631) (Data S1).

Table 3 shows the prognostic factors for RFS. In univariate analysis, tumor grade (G3) (*p* = 0.003), introduction of POT (*p* = 0.016), higher number of liver metastases (*p* < 0.001), R1 status (*p* = 0.011), and microwave ablation (*p* = 0.014) were risk factors for shorter RFS. Multivariate analysis showed that tumor grade (G3) (*p* = 0.007; hazard ratio = 3.1, 95% CI: 1.4–7.2) and higher number of metastatic liver metastases (*p* < 0.001; hazard ratio = 1.1, 95% CI: 1.0–1.1) were associated with poor RFS.

**TABLE 3:** Prognostic factors for recurrence free survival in patients with R0/1 resection (*n* = 44).

Prognostic factor	Univariate analysis		Multivariate analysis	
	Hazard ratio (95% CI)	*p*	Hazard ratio (95% CI)	*p*
Gender (male)	0.9 (0.5–1.7)	0.705		
Age	1.0 (0.9–1.0)	0.219		
Location of primary tumor (pancreas)	0.7 (0.3–1.4)	0.269		
Tumor grade (G3)	3.5 (1.5–8.1)	0.003*	3.1 (1.4–7.2)	0.007*
Maximal size of liver tumor ≥ 30 mm	0.8 (0.4–1.6)	0.551		
Synchronous liver metastasis	0.7 (0.4–1.4)	0.379		
Concomitant liver resection	1.1 (0.6–2.1)	0.736		
Preoperative therapy (yes)	2.3 (1.2–4.7)	0.016*		0.102
Number of liver metastases	1.1 (1.0–1.1)	< 0.001*	1.1 (1.0–1.1)	< 0.001*
Surgical status (R1)	3.9 (1.4–11.3)	0.011*		0.270
Microwave ablation (yes)	2.7 (1.2–5.8)	0.014*		0.339
Adjuvant therapy (yes)	1.2 (0.6–2.2)	0.635		

Abbreviation: CI, confidence interval.

* *p* < 0.05 was considered statistically significant.

### 3.4 Cut‐Off Value for the Number of Liver Metastases Associated With Poor RFS

**FIGURE 3:**
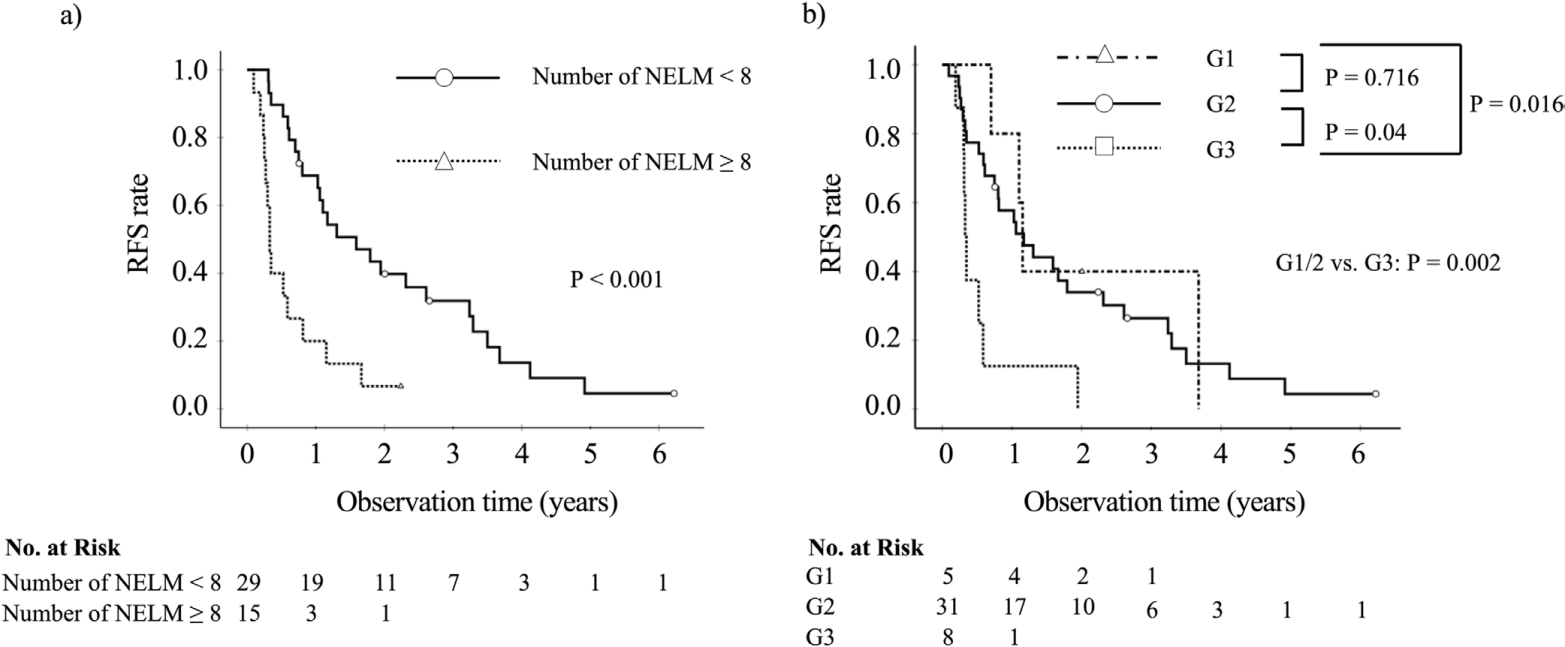
Comparison of RFS by the number of NELM (a) and tumor grade (b). (a) Patients with ≥ 8 NELM had poorer RFS than those with < 8 NELM. (b) Patients with NET G1 and G2 had comparable RFS, however, those with NET G3 had significantly poorer RFS.

The optimal cut‐off value for the number of liver metastases for RFS was eight as determined by the minimum *p*‐value approach (Supplementary S2). The cut‐off value calculated by X‐tile also showed the same cut‐off value (data not shown). The Kaplan–Meier survival curve of each prognostic factor for RFS was shown in Figure 3. For patients with < 8 liver metastases and those with ≥ 8 liver metastases, the median RFS time was 19.1 and 3.9 months, respectively (*p* < 0.001). Although there was no significant difference between NET‐G1 and NET‐G2 patients (*p* = 0.716), NET‐G3 patients had significantly shorter RFS compared to both NET‐G1 (*p* = 0.016) and NET‐G2 (*p* = 0.04) patients. The median RFS time for patients with NET‐G1, G2, and G3 was 13.8, 14.0, and 3.9 months, respectively. In the multivariate analysis including the number of liver metastases as a categorical value (≥ 8 or < 8), tumor grade (G3) (*p* = 0.003; hazard ratio = 3.6, 95% CI: 1.5–8.4), and the number of metastatic liver metastases ≥ 8 (*p* < 0.001; hazard ratio = 3.8, 95% CI: 1.8–7.9) were independent factors associated with poor RFS (Table 4).

**TABLE 4:** Prognostic factors for recurrence free survival in patients with R0/1 resection (*n* = 44); number of liver metastases was shown as a categorical value (≥ 8 or < 8).

Prognostic factor	Univariate analysis		Multivariate analysis	
	Hazard ratio (95% CI)	*p*	Hazard ratio (95% CI)	*p*
Gender (male)	0.9 (0.5–1.7)	0.705		
Age	1.0 (0.9–1.0)	0.219		
Location of primary tumor (pancreas)	0.7 (0.3–1.4)	0.269		
Tumor grade (G3)	3.5 (1.5–8.1)	0.003*	3.6 (1.5–8.4)	0.003*
Maximal size of liver tumor ≥ 30 mm	0.8 (0.4–1.6)	0.551		
Synchronous liver metastasis	0.7 (0.4–1.4)	0.379		
Concomitant liver resection	1.1 (0.6–2.1)	0.736		
Preoperative therapy (yes)	2.3 (1.2–4.7)	0.016*		0.193
Number of liver metastases ≥ 8	3.7 (1.8–7.7)	< 0.001*	3.8 (1.8–7.9)	< 0.001*
Surgical status (R1)	3.9 (1.4–11.3)	0.011*		0.149
Microwave ablation (yes)	2.7 (1.2–5.8)	0.014*		0.732
Adjuvant therapy (yes)	1.2 (0.6–2.2)	0.635		

Abbreviation: CI, confidence interval.

* *p* < 0.05 was considered statistically significant.

## 4 Discussion

This study of NENs from a high‐volume center found that curative surgical resection was associated with prolonged OS after adjustment for tumor grade. A greater number of NELM and NET‐G3 were associated with poor RFS; the cut‐off value for the number of NELM was eight. The very short median duration of RFS for patients with NET‐G3 or those with NELM ≥ 8 (3.9 months) suggests that resection should be avoided in such patients. On the other hand, considering the median RFS for patients with NET‐G1/2 (13.8 months) or those with NELM < 8 (19.1 months) was relatively long, these patients are good candidates for resection for those needing a break from systemic therapy. While OS was set as the primary outcome of this study, we also emphasized RFS as a clinically relevant secondary outcome. Because OS can be affected by various post‐recurrence treatments, RFS provides a more direct measure of the initial benefit of surgery and may help refine patient selection.

Previous studies have reported that surgical resection for NELM improved OS with MST ranging from 81 to 126 months [6]. The patients with R0/1 resection in the present study showed the MST of 13.6 years, which was comparable to these studies. The patients with R2 resection also had better outcomes than those in the medication group. However, our study did not aim to determine the effect of R2 resections. The R2 resections in our study were not planned for surgical debulking, but were failed R0/1 resections due to an unexpectedly large number of liver tumors. Therefore, our findings do not necessarily support the cytoreductive surgery that was investigated by some previous studies [8, 10, 26].

Another important goal of surgical resection for patients with NELM is to provide a longer RFS as well as cure or longer OS. Patients with NENs have been reported to have a long treatment period because of the relatively low aggressiveness of the tumors. The side effects and costs of anti‐tumor drugs are a continuing burden on patients [27, 28]. Therefore, surgical resection can provide patients a reprieve from systemic therapy during the recurrence free period. However, a short RFS following curative resection for NELM would argue against the value of surgery. In the present study, 22 of 44 patients (50%) who underwent curative resection developed recurrence within 1 year after surgery and the median RFS time was 1 year. This is consistent with previous reports [12, 13, 14, 15, 16]. Despite the fact that most patients relapse within 2 years after curative resection for NELM, the National Comprehensive Cancer Network [29], European Neuroendocrine Tumor Society [30], and Japanese Neuroendocrine Tumor Society [31] guidelines recommend surgical resection for NELM when complete resection is possible. However, given the heterogeneous biology of NETs, it is necessary to define surgical indications that are objective and include biological factors associated with longer RFS for patients with NELM.

Previous studies have reported the risk factors for recurrence, namely, timing of NELM occurrence [20], pancreatic NENs [22], tumor differentiation [17], non‐functional tumors [21], lymph node metastasis [21, 22], a step‐up of Ki‐67 index in metastases compared to primary tumor [19]{Lv, 2019 no. 41} and microscopic positive surgical margins [13, 22]. Most of these studies were retrospective, multi‐institutional studies owing to the rarity of NENs, which led to inconsistent treatment and follow‐up protocols. Furthermore, four of these multicenter studies were conducted in the same cohort [17, 20, 21, 22], therefore there are not enough published data to suggest risk factors for poor RFS.

Masui et al. [15] evaluated 26 pancreatic NET patients with liver metastases who underwent R0/1 resection and found that Ki‐67 index > 5% and the number of NELM ≥ 7 were the risk factors for predicting short RFS. The results were generally consistent with our report of 44 gastroenteropancreatic NET patients with liver metastases who underwent R0/1 resection. The median number of liver metastases in that study was only 2.5. The median number of liver metastases in the present study was 8. Additionally our analysis included patients who had not undergone resection, which means that a broader population analysis was performed in our study.

The number of liver metastases was associated with RFS in NELM patients not only because of the aggressiveness of the tumor, but also because of the detection limitations of preoperative imaging. Elias et al. [32] investigated the accuracy of preoperative imaging compared with thin‐slice pathology and reported that the accuracy was 24% for somatostatin receptor scintigraphy, 38% for computed tomography and ultrasonography, and 49% for magnetic resonance imaging. The authors concluded that 50% of NELM were undetectable on preoperative imaging. Although we generally used a combination of ultrasonography, contrast‐enhanced computed tomography, gadoxetic acid‐enhanced magnetic resonance imaging, and somatostatin receptor scintigraphy for preoperative imaging, the percentage of cases in which the number of liver metastases on pathology exceeded the preoperative imaging diagnosis was 9% for cases with < 8 liver metastases and 64% for cases with ≥ 8 liver metastases in our cohort. In other words, it suggests that some tumors may have gone undetected in patients with ≥ 8 liver metastases, resulting in incomplete resection.

Although the number of NELM ≥ 8 was a risk factor for shorter RFS and recurrence within 1 year following surgical resection led to poor OS, the number of NELM ≥ 8 was not associated with OS (MST: 13.6 years vs. 11.4 years for < 8 vs. ≥ 8 liver metastases, respectively; *p* = 0.839). This is because the progression of NENs is very slow and multidisciplinary treatments have been introduced to address recurrence. It is sometimes difficult to clarify the association between specific factors and OS in patients with NENs [18].

Tumor grade (G3) was also independently associated with poor RFS in the present study. Few studies have distinguished NET‐G3 from NEC [15, 33], and no study has shown the NET‐G3 as an independent prognostic factor for patients with NELM because NET‐G3 was only recently defined. Additionally, the number of patients in that study was very small, so statistical inference was difficult [15]. Our study included 23 patients with NET‐G3, 14 of whom underwent surgical resection, and showed that NET‐G3 was associated with poor OS and RFS.

While POT is commonly introduced prior to surgical resection for metastatic tumors in other cancers, POT was not associated with prolonged RFS for NELM patients in the multivariate analysis of this study. Furthermore, the median RFS time of the patients with POT was shorter than that of patients without POT in univariate analysis (7.0 vs. 19.1 months: *p* = 0.016). There are two possible reasons for this result: one is selection bias for POT administration, and the other is inadequate efficacy of anti‐tumor drugs; sunitinib [34] and everolimus were mainly used for POT, with their historical response rates ranging from 5% to 10% [27, 28].

This study had several limitations. First, because this was a single‐institution study, the number of patients was relatively small compared with that in multi‐institution or population‐based studies. Second, this is a retrospective study and may be subject to selection bias. For example, “surgical status” (R0/1 resection, R2 resection, or medication) was included as a variable in the multivariate analysis; however, it is influenced by preoperative patient and tumor characteristics that determine surgical eligibility. In addition, in the medication group, primary tumor resection was rarely performed (39%), and these patients generally had a larger overall tumor burden, which may have contributed to their poorer prognosis compared with the R0/1 resection group. This imbalance is inherent to the retrospective design of the study and could not be fully adjusted for in the analysis. Therefore, the results should be interpreted with caution and not as evidence of the superiority of one treatment over another. This selection bias may be the reason why the patients with R2 resection showed a better prognosis than those who received medication in the Cox's proportional hazard model analysis. However, data for all consecutive patients during the study period were analyzed, and our institutional database is maintained prospectively. Therefore, we believe that the influence of selection bias was minimized in this study. Third, 68Ga DOTATOC‐PET/CT was not performed as a preoperative imaging because it was not covered by Japanese health insurance. Fourth, the number of liver metastases was determined based on pathological examination. We adopted pathological findings because the accuracy of radiological assessment may vary depending on the imaging modalities and their resolution available at the time of diagnosis. In addition, the Ki‐67 index was also assessed based on pathological examination, by evaluating both primary and metastatic lesions and adopting the highest Ki‐67 value among them. However, neither the exact number of metastases nor the maximal Ki‐67 index can be determined preoperatively. Therefore, to improve clinical applicability, future prospective studies using high‐resolution preoperative imaging and predictive strategies are needed. To address these limitations, a multi‐institutional study with a large number of patients is required, and better treatment strategies should be considered, especially for patients with ≥ 8 liver metastases or NET‐G3, in combination with POT or with postoperative adjuvant therapy, including PRRT. Moreover, advances in basic research have revealed biomarkers for NENs, and indications for surgery could include liquid biopsy, such as the NETest [35].

## 5 Conclusions

This study suggests that surgical resection improves the OS in patients with NELM. Although the high recurrence rate even after curative surgical resection is an issue that needs to be solved, this study also suggests that fewer than 8 liver metastases and NET‐G1/2 are good indications for surgical resection in patients with NELM considering the longer RFS. In contrast, surgical resection for patients with 8 or more liver metastases or NET‐G3 requires deliberate selection.

## Author Contributions

**Daisuke Asano:** conceptualization, methodology, data curation, investigation, formal analysis, funding acquisition, writing – original draft, project administration. **Toshitaka Sugawara:** writing – review and editing, supervision, software, methodology. **Keiichi Akahoshi:** writing – review and editing, supervision, investigation. **Shotaro Gan:** investigation, data curation. **Shohei Motohashi:** data curation, investigation. **Shuichi Watanabe:** conceptualization, investigation, methodology, data curation. **Yoshiya Ishikawa:** writing – review and editing, supervision. **Hiroki Ueda:** supervision, writing – review and editing. **Atsushi Kudo:** conceptualization, writing – review and editing, supervision. **Daisuke Ban:** supervision, writing – review and editing.

## Ethics Statement

All study procedures were approved by our institutional review board (Human Research Ethics Committee, Institute of Science Tokyo; approval ID: 1080).

## Conflicts of Interest

None of the authors have personal conflicts of interest associated with the publication of this article. D. Ban is an editorial member of Annals of Gastroenterological Surgery.

## Supporting information


**Data S1:** Kaplan–Meier curves for OS in patients with recurrence within 1 year and after 1 year and in patients with R2 resection and no recurrence. Patients with R2 resection and those with recurrence within 1 year had a similar poor prognosis.

**Data S2:** A graph showing *p* values corresponding to each cut‐off value of NELM for RFS. According to the minimum *p* value approach, the best cut‐off value was eight.
