# Supplementary figures and images for: Indications for Surgical Resection in Patients With Neuroendocrine Tumor Liver Metastases: An Intensive Surgical Experience of a High‐Volume Center
Source: Ann Gastroenterol Surg. 2025 Aug 27;10(1):219–28. doi: 10.1002/ags3.70082 (PMC12757151; doi:10.1002/ags3.70082)

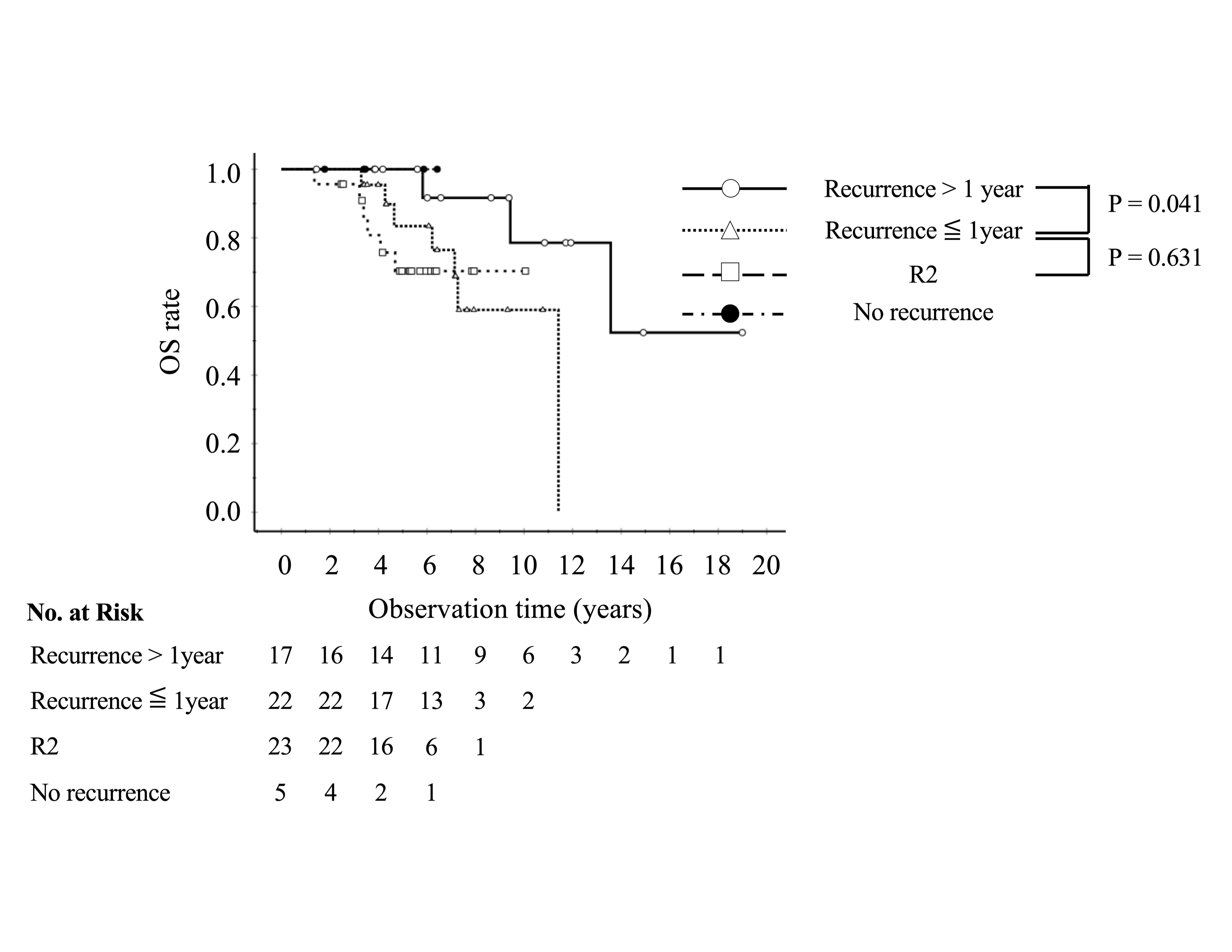

Supplement: Supplementary file 1 — Data S1: Kaplan–Meier curves for OS in patients with recurrence within 1 year and after 1 year and in patients with R2 resection and no recurrence. Patients with R2 resection and those with recurrence within 1 year had a similar poor prognosis. [file AGS3-10-219-s002.tiff]

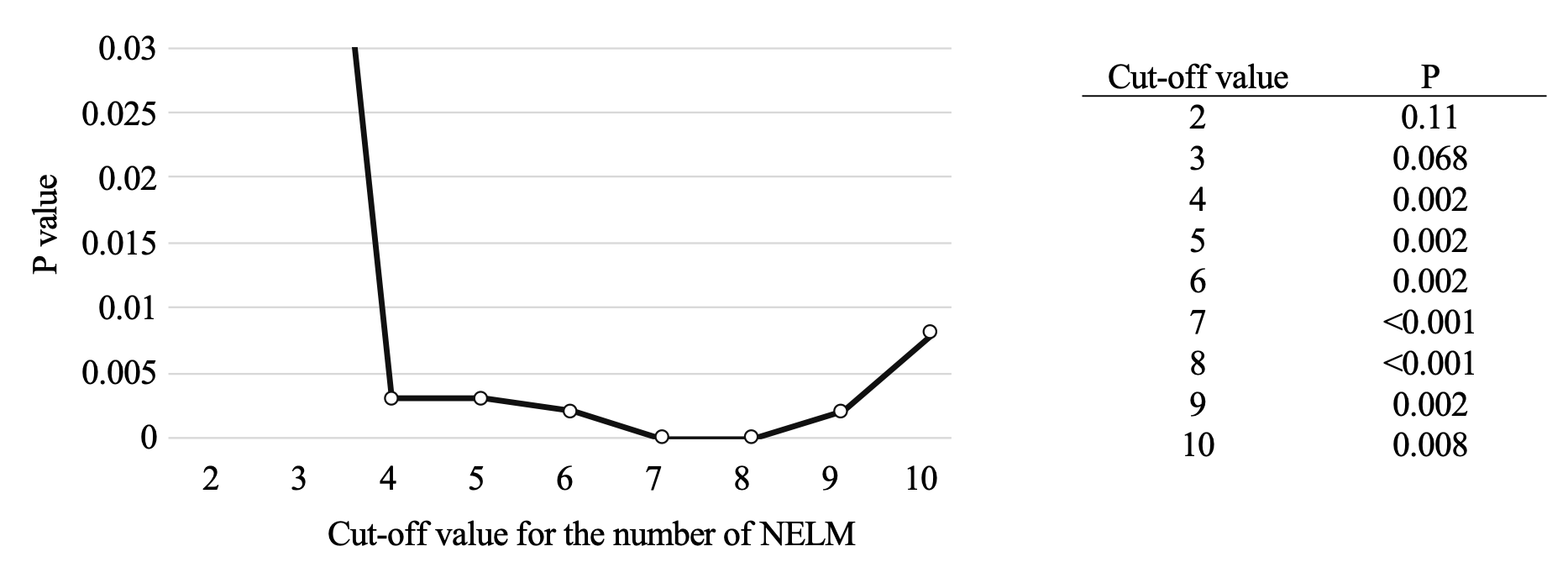

Supplement: Supplementary file 2 — Data S2: A graph showing p values corresponding to each cut‐off value of NELM for RFS. According to the minimum p value approach, the best cut‐off value was eight. [file AGS3-10-219-s001.tiff]
